# Quantum Fourier transform is the building block for creating entanglement

**DOI:** 10.1038/s41598-021-01745-x

**Published:** 2021-11-15

**Authors:** Mario Mastriani

**Affiliations:** Knight Foundation School of Computing & Information Sciences, Florida International University, 11200 S.W. 8th Street, Miami, FL 33199 USA

**Keywords:** Physics, Quantum physics

## Abstract

This study demonstrates entanglement can be exclusively constituted by quantum Fourier transform (QFT) blocks. A bridge between entanglement and QFT will allow incorporating a spectral analysis to the already traditional temporal approach of entanglement, which will result in the development of new more performant, and fault-tolerant protocols to be used in quantum computing as well as quantum communication, with particular emphasis in the future quantum Internet.

## Introduction

Jean-Baptiste Joseph Fourier (Auxerre, France, 21 March 1768, Paris, 16 May 1830) was a French mathematician and physicist, a disciple of Joseph-Louis Lagrange (Turin, Italy, 25 January 1736, Paris, 10 April 1813), known for his work on the decomposition of periodic functions into convergent trigonometric series called Fourier series, a method with which he managed to solve the heat equation. The projection of his work in the two centuries following his death on areas as diverse as electricity, optics, electronics, and so on, culminated during the twentieth century in the creation of the famous Discrete Fourier Transform^1^, Fast Fourier Transform^2^, and Quantum Fourier Transform^3^ (QFT), where the latter constitutes a key piece within Quantum Information Processing^4^ for the case of those quantum algorithms that require a phase estimation^5^, or phase estimation in qudit systems^6^, as well as, the presence of QFT in a *d*-level quantum system^7^.

On the other hand, entanglement^8–10^, so reviled by Albert Einstein, Boris Podolsky, and Nathan Rosen in their so famous 1935 paper^11^, has become the cornerstone of Quantum Computing^4^ and Quantum Communication^12^, in particular, in communications protocols such as quantum teleportation^13^, quantum secret sharing^14^, quantum key distribution^15^, quantum secure direct communication^16^, and quantum repeaters^17^, with a marked commitment to the future quantum Internet^18–22^.

The union of both entities, i.e. QFT, and entanglement, seems at first something quite strange, at least in the way it is presented in this work, where the first becomes a basal element for the creation of the second, however, the approach that will be presented then it will allow access to the hidden face of the entanglement, its spectral face.

QFT is constituted by an important family of quantum operations over the ring ℤ_2_^n^. The *n*-qubit QFT makes a coherent mapping from an input state or qubit string $\left| x \right\rangle = \left| {x_{1} \ldots x_{n} } \right\rangle$ to an output state or qubit string $\left| y \right\rangle = \left| {y_{1} \ldots y_{n} } \right\rangle$ in the computational basis^23^ as follows:

1 $$ \left| y \right\rangle \mapsto \frac{1}{{\sqrt {2^{n} } }}\sum\limits_{v = 0}^{{2^{n} - 1}} {\omega_{{2^{n} }}^{u.v} } \left| x \right\rangle ,\;\;\;\;\;\;\;\;\;\;\;\;u = 0,\;1,\;2,\; \ldots ,\;2^{n} - 1 $$

where $\omega_{{2^{n} }} = e^{{i2\pi /2^{n} }}$ is the 2^*n*^ root of unity, while the inverse QFT is:

2 $$ \left| x \right\rangle \mapsto \frac{1}{{\sqrt {2^{n} } }}\sum\limits_{u = 0}^{{2^{n} - 1}} {\omega_{{2^{n} }}^{ - v.u} } \left| y \right\rangle ,\;\;\;\;\;\;\;\;\;\;\;\;v = 0,\;1,\;2,\; \ldots ,\;2^{n} - 1 $$

The Hadamard matrix *H* is equivalent to the 1-qubit QFT and its inverse^24,25^,

3 $$ F_{{2^{1} }} = H = \frac{1}{\sqrt 2 }\left[ {\begin{array}{*{20}c} 1 & {\;\;1} \\ 1 & { - 1} \\ \end{array} } \right] = H^{ - 1} = F_{{2^{1} }}^{ - 1} \in {\mathbb{C}}^{{2^{1} \times 2^{1} }} . $$

That is, for the 1-qubit QFT all its components are equivalent. Instead, for the 2-qubit QFT, the same does not happen, since

4a $$ F_{{2^{2} }} = \frac{1}{2}\left[ {\begin{array}{*{20}c} 1 & 1 & 1 & 1 \\ 1 & i & { - 1} & { - i} \\ 1 & { - 1} & 1 & { - 1} \\ 1 & { - i} & { - 1} & i \\ \end{array} } \right], $$

and

4b $$ F_{{2^{2} }}^{ - 1} = \frac{1}{2}\left[ {\begin{array}{*{20}c} 1 & 1 & 1 & 1 \\ 1 & { - i} & { - 1} & i \\ 1 & { - 1} & 1 & { - 1} \\ 1 & i & { - 1} & { - i} \\ \end{array} } \right], $$

are different: $F_{{2^{2} }} \ne F_{{2^{2} }}^{ - 1}$, where $F_{{2^{2} }} \wedge F_{{2^{2} }}^{ - 1} \in {\mathbb{C}}^{{2^{2} \times 2^{2} }}$. On the other hand, the Feynman’s gate^4^ (also known as *Controlled*-X, CNOT, or CX gate), as well as its flipped version are respectively:

5a $$ CNOT = \left[ {\begin{array}{*{20}c} 1 & 0 & 0 & 0 \\ 0 & 1 & 0 & 0 \\ 0 & 0 & 0 & 1 \\ 0 & 0 & 1 & 0 \\ \end{array} } \right], $$

and

5b $$ CNOT_{flipped} = \left[ {\begin{array}{*{20}c} 1 & 0 & 0 & 0 \\ 0 & 0 & 0 & 1 \\ 0 & 0 & 1 & 0 \\ 0 & 1 & 0 & 0 \\ \end{array} } \right], $$

where the difference between them consists in that in Eq. (5a) the upper qubit is the control qubit, while the lower qubit is the target qubit. Instead, in the version of Eq. (5b) it is exactly the opposite, being: $CNOT_{flipped} = \left( {H \otimes H} \right) \times CNOT \times \left( {H \otimes H} \right)$, “×” the matrix product, and “⊗” the Kronecker product^4^.

Multiplying both $F_{{2^{2} }}$ by itself and $F_{{2^{2} }}^{ - 1}$ by itself, both multiplications result equal to the *CNOT*_flipped_ gate of Eq. (5b): $F_{{2^{2} }} \times F_{{2^{2} }} = F_{{2^{2} }}^{ - 1} \times F_{{2^{2} }}^{ - 1} = CNOT_{flipped}$. This can be easily verified by multiplying *CNOT*_flipped_ by itself, and $F_{{2^{2} }} \times F_{{2^{2} }}$ by $F_{{2^{2} }}^{ - 1} \times F_{{2^{2} }}^{ - 1}$ and regrouping,

6 $$ \begin{gathered} CNOT_{flipped} \times CNOT_{flipped} = \left( {F_{{2^{2} }} \times F_{{2^{2} }} } \right) \times \left( {F_{{2^{2} }}^{ - 1} \times F_{{2^{2} }}^{ - 1} } \right) \hfill \\ \quad \quad \quad \quad \quad \quad \quad \quad \quad \;\;\,{\kern 1pt} = F_{{2^{2} }} \times \left( {F_{{2^{2} }} \times F_{{2^{2} }}^{ - 1} } \right) \times F_{{2^{2} }}^{ - 1} = F_{{2^{2} }} \times I \times F_{{2^{2} }}^{ - 1} = F_{{2^{2} }} \times F_{{2^{2} }}^{ - 1} = I_{{2^{2} \times 2^{2} }} . \hfill \\ \end{gathered} $$

However,

7a $$ \sqrt {CNOT} = \left[ {\begin{array}{*{20}c} 1 & 0 & 0 & 0 \\ 0 & 1 & 0 & 0 \\ 0 & 0 & {\left( {1 + i} \right)/2} & {\left( {1 - i} \right)/2} \\ 0 & 0 & {\left( {1 - i} \right)/2} & {\left( {1 + i} \right)/2} \\ \end{array} } \right], $$

and

7b $$ \sqrt {CNOT_{flipped} } = \left[ {\begin{array}{*{20}c} 1 & 0 & 0 & 0 \\ 0 & {\left( {1 + i} \right)/2} & 0 & {\left( {1 - i} \right)/2} \\ 0 & 0 & 1 & 0 \\ 0 & {\left( {1 - i} \right)/2} & 0 & {\left( {1 + i} \right)/2} \\ \end{array} } \right], $$

Therefore, $\sqrt {CNOT} \ne F_{{2^{2} }}$ and $\sqrt {CNOT_{flipped} } \ne F_{{2^{2} }}$. Finally, the *CNOT* gate is equal to the flipped version of the multiplication of QFT $F_{{2^{2} }}$ by itself,

8 $$ \left( {H \otimes H} \right) \times \left( {F_{{2^{2} }} \times F_{{2^{2} }} } \right) \times \left( {H \otimes H} \right) = \left( {H \otimes H} \right) \times \left( {F_{{2^{2} }}^{ - 1} \times F_{{2^{2} }}^{ - 1} } \right) \times \left( {H \otimes H} \right) = CNOT. $$

Equation (8) is fundamental in the creation of the entanglement for two or more qubits, as well as in all the applications that require it, as is the case of quantum teleportation^11^.

## Bell states

Pauli’s matrices^4^ can be expressed in terms of the so-named Hadamard rotation gates^26^ or the general unitary operator $$U\left( {\theta ,\varphi ,\lambda } \right) = \left[ {\begin{array}{*{20}l} {{\text{cos}}\left( {\theta /2} \right)} \hfill & { - {\text{e}}^{i\,\lambda } {\text{sin}}\left( {\theta /2} \right)} \hfill \\ {{\text{e}}^{i\varphi } {\text{sin}}\left( {\theta /2} \right)} \hfill & {{\text{e}}^{{i\left( {\lambda + \varphi } \right)}} {\text{cos}}\left( {\theta /2} \right)} \hfill \\ \end{array} } \right]$$ as follows:

9a $$ I = H_{{\text{I}}} H_{{\text{I}}} = H_{{{\text{III}}}} H_{{{\text{III}}}} = H_{{{\text{II}}}} H_{{{\text{IV}}}} = H_{{{\text{IV}}}} H_{{{\text{II}}}} , $$

9b $$ X = H_{{{\text{III}}}} H_{{{\text{II}}}} = H_{{{\text{II}}}} H_{{\text{I}}} = H_{{\text{I}}} H_{{{\text{IV}}}} = H_{{{\text{IV}}}} H_{{{\text{III}}}} , $$

9c $$ Y = iH_{{{\text{III}}}} H_{{\text{I}}} = iH_{{{\text{II}}}} H_{{{\text{II}}}} = \, - iH_{{{\text{IV}}}} H_{{{\text{IV}}}} = \, - iH_{{\text{I}}} H_{{{\text{III}}}} ,{\text{ and}} $$

9d $$ Z = \, - H_{{{\text{II}}}} H_{{{\text{III}}}} = H_{{\text{I}}} H_{{{\text{II}}}} = \, - H_{{{\text{III}}}} H_{{{\text{IV}}}} = H_{{{\text{IV}}}} H_{{\text{I}}} , $$

where *I* is a 2 × 2 identity matrix, $i = \sqrt { - 1}$, $H_{I} = H = U\left( {\pi /2,0,\pi } \right)$ of Eq. (3), while

10 $$ H_{II} = \frac{1}{\sqrt 2 }\left[ {\begin{array}{*{20}c} 1 & { - 1} \\ 1 & 1 \\ \end{array} } \right] = U\left( {\pi /2,0,0,} \right),\;\;\;H_{III} = \frac{1}{\sqrt 2 }\left[ {\begin{array}{*{20}c} { - 1} & 1 \\ 1 & 1 \\ \end{array} } \right] = U\left( {5\pi /2,\pi ,0} \right)\;\;{\text{and}}\;\;H_{IV} = \frac{1}{\sqrt 2 }\left[ {\begin{array}{*{20}c} 1 & 1 \\ { - 1} & 1 \\ \end{array} } \right] = U\left( {\pi /2,\pi ,\pi } \right) $$

**Figure 1:**
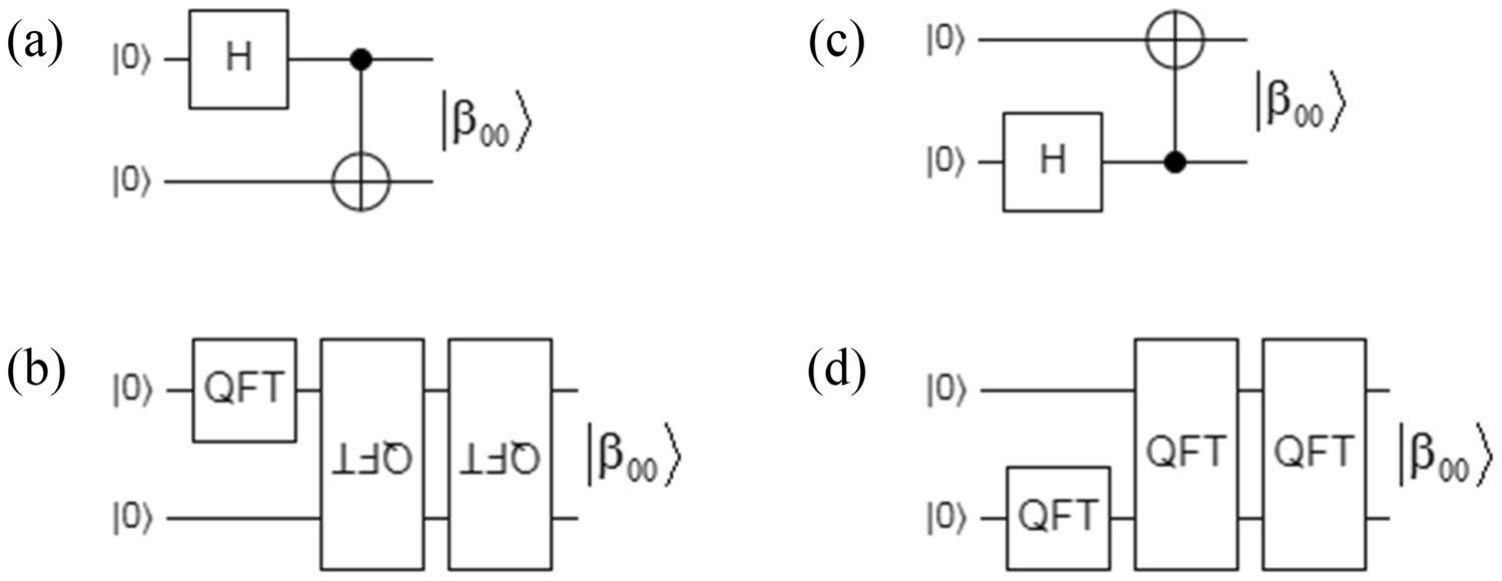
Representation of the $\left| {\beta_{00} } \right\rangle = \left| {\Phi^{ + } } \right\rangle$ Bell state in terms of QFT: (**a**) original version based on H and CNOT gates, (**b**) its representations in terms of one QFT_2_^1^_×2_^1^ and two flipped QFT_2_^2^_×2_^2^, (**c**) original version with one H and one flipped CNOT gates, and (**d**) its representations in terms of one QFT_2_^1^_×2_^1^ and two QFT_2_^2^_×2_^2^.

The equivalence of Eq. (9b) can be used to represent the four Bell states:

11a $$ \left| {\beta_{00} } \right\rangle = \left| {\Phi^{ + } } \right\rangle = 1/\sqrt 2 \left( {\left| {00} \right\rangle + \left| {11} \right\rangle } \right) = CNOT\left( {H \otimes I} \right)\left| {00} \right\rangle , $$

11b $$ \left| {\beta_{01} } \right\rangle = \left| {\Psi^{ + } } \right\rangle = 1/\sqrt 2 \left( {\left| {01} \right\rangle + \left| {10} \right\rangle } \right) = CNOT\left( {H \otimes I} \right)\left| {01} \right\rangle = CNOT\left( {H \otimes I} \right)\left| 0 \right\rangle X\left| 0 \right\rangle , $$

11c $$ \left| {\beta_{10} } \right\rangle = \left| {\Phi^{ - } } \right\rangle = 1/\sqrt 2 \left( {\left| {00} \right\rangle - \left| {11} \right\rangle } \right) = CNOT\left( {H \otimes I} \right)\left| {10} \right\rangle = CNOT\left( {H \otimes I} \right)X\left| 0 \right\rangle \left| 0 \right\rangle ,\;\;{\text{and}} $$

11d $$ \left| {\beta_{11} } \right\rangle = \left| {\Psi^{ - } } \right\rangle = 1/\sqrt 2 \left( {\left| {01} \right\rangle - \left| {10} \right\rangle } \right) = CNOT\left( {H \otimes I} \right)\left| {11} \right\rangle = CNOT\left( {H \otimes I} \right)X\left| 0 \right\rangle X\left| 0 \right\rangle , $$

with $$\left| 0 \right\rangle = \left[ {\begin{array}{*{20}c} 1 \\ 0 \\ \end{array} } \right]$$, and $$\left| 1 \right\rangle = \left[ {\begin{array}{*{20}c} 0 \\ 1 \\ \end{array} } \right]$$. That is, replacing in Eqs. (11a–11d) the equivalence corresponding to *X* of Eq. (9b),

*H* of Eq. (3), *I* of Eq. (9a), and *CNOT* of Eq. (8), it is possible to implement the four Bell states exclusively basing on QFT blocks. Without losing generality, Fig. 1a,c represents the implementation of the $\left| {\beta_{00} } \right\rangle = \left| {\Phi^{ + } } \right\rangle$ Bell state in terms of its two original versions (direct and flipped), while Fig. 1c,d constitute their respective counterparts based exclusively on QFT blocks, confirming that these blocks are all that is needed to create entanglement while revealing its spectral nature.

## N-qubits Greenberger–Horne–Zeilinger (GHZ_N_) states

This family of configurations is the most commonly used in practice when it comes to entanglement between three or more particles^4,8–10^, being its general form as follows:

12 $$ \left| {GHZ_{N} } \right\rangle = 1/\sqrt 2 \left( {\left| 0 \right\rangle^{ \otimes N} + \left| 1 \right\rangle^{ \otimes N} } \right) $$

Without loss of generality, in this study only $\left| {GHZ_{3} } \right\rangle$ and $\left| {GHZ_{4} } \right\rangle$ are implemented in terms of QFT blocks, where:

13 $$ \left| {GHZ_{3} } \right\rangle = 1/\sqrt 2 \left( {\left| {000} \right\rangle + \left| {111} \right\rangle } \right) = \left( {I_{2 \times 2} \otimes CNOT} \right)\left( {CNOT \otimes I_{2 \times 2} } \right)\left( {H \otimes I_{4 \times 4} } \right)\left| {000} \right\rangle ,\;{\text{and}} $$

14 $$ \left| {GHZ_{4} } \right\rangle = 1/\sqrt 2 \left( {\left| {0000} \right\rangle + \left| {1111} \right\rangle } \right) = \left( {I_{4 \times 4} \otimes CNOT} \right)\left( {I_{2 \times 2} \otimes CNOT \otimes I_{2 \times 2} } \right)\left( {CNOT \otimes I_{4 \times 4} } \right)\left( {H \otimes I_{8 \times 8} } \right)\left| {0000} \right\rangle . $$

**Figure 2:**
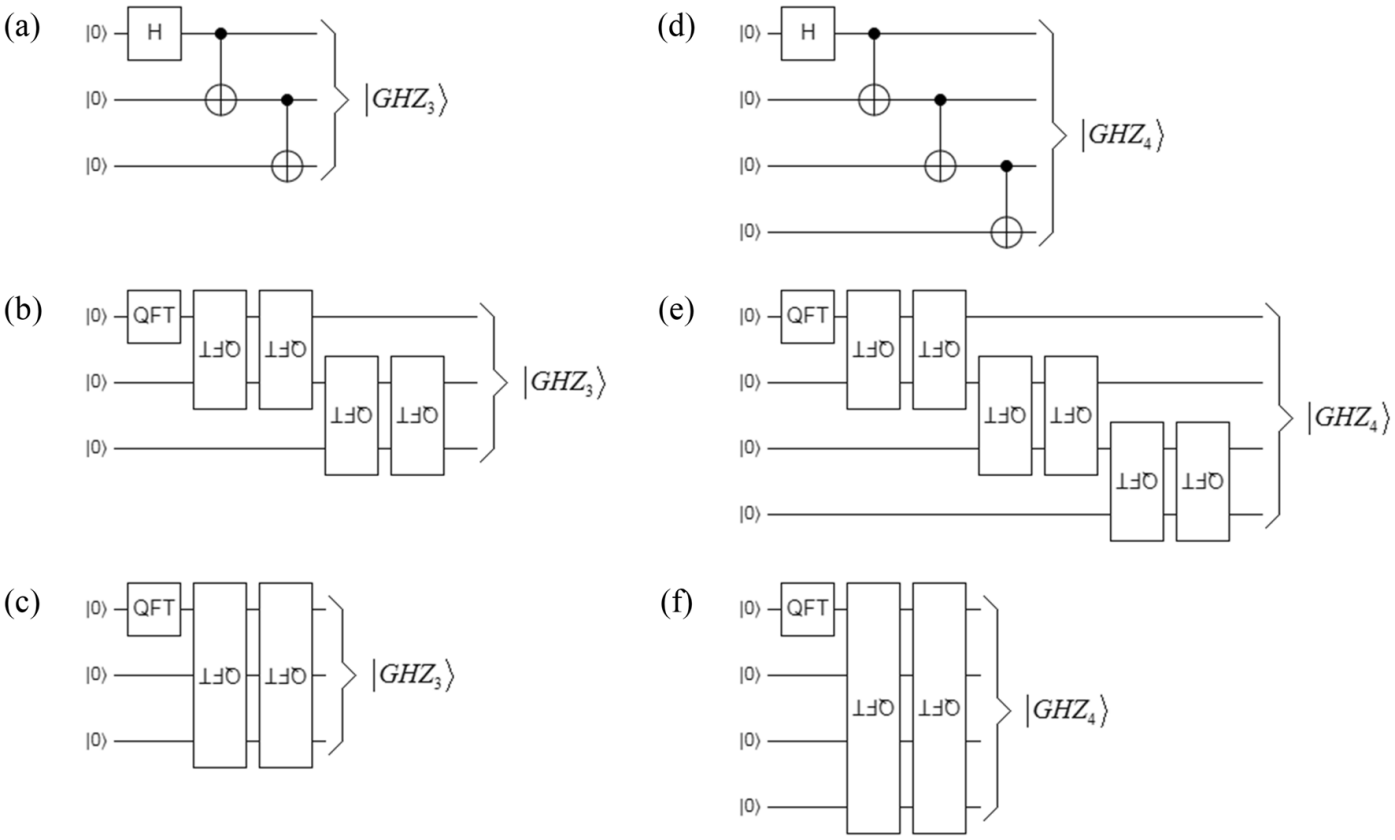
Implementations of $\left| {GHZ_{3} } \right\rangle$ and $\left| {GHZ_{4} } \right\rangle$: (**a**) original version of $\left| {GHZ_{3} } \right\rangle$ in terms of one *H* and two CNOT gates, (**b**) $\left| {GHZ_{3} } \right\rangle$ thanks to one QFT_2_^1^_×2_^1^ and four flipped QFT_2_^2^_×2_^2^, (**c**) $\left| {GHZ_{3} } \right\rangle$ based on one QFT_2_^1^_×2_^1^ and two flipped QFT_2_^3^_×2_^3^, (**d**) original version of $\left| {GHZ_{4} } \right\rangle$ in terms of one *H* and three CNOT gates, (**e**) $\left| {GHZ_{4} } \right\rangle$ thanks to one QFT_2_^1^_×2_^1^ and six flipped QFT_2_^2^_×2_^2^, (**f**) $\left| {GHZ_{4} } \right\rangle$ based on one QFT_2_^1^_×2_^1^ and two flipped QFT_2_^4^_×2_^4^.

Equations (13) and (14) are graphically represented in Fig. 2a and d, respectively. Figure 2b shows $\left| {GHZ_{3} } \right\rangle$ with one QFT_2_^1^_×2_^1^ and four flipped QFT_2_^2^_×2_^2^, while Fig. 2c represents it thanks to one QFT_2_^1^_×2_^1^ and two flipped QFT_2_^3^_×2_^3^. Finally, Fig. 2e shows $\left| {GHZ_{4} } \right\rangle$ with one QFT_2_^1^_×2_^1^ and six flipped QFT_2_^2^_×2_^2^, while Fig. 2f represents it thanks to one QFT_2_^1^_×2_^1^ and two flipped QFT_2_^4^_×2_^4^.

Equivalences between Fig. 2a–c as well as between Fig. 2d–f, show again the spectral nature of the entanglement even for the case of more than two particles entangled at the same time. In fact, the equivalences present in Fig. 2 between (*N* − 1) pairs of flipped QFT_2_^2^_×2_^2^ and two flipped QFT_2_^N^_×2_^N^, show that the equivalence of Fig. 1 is not a simple coincidence for a particular case like a Bell state, but actually, the entanglement in all its manifestations has a spectral nature, where the QFT is the essential instrument for a spectral tomography of it. It only remains to project this equivalence, in perhaps the most conspicuous application of entanglement, quantum teleportation^13^.

## Teleportation

**Figure 3:**
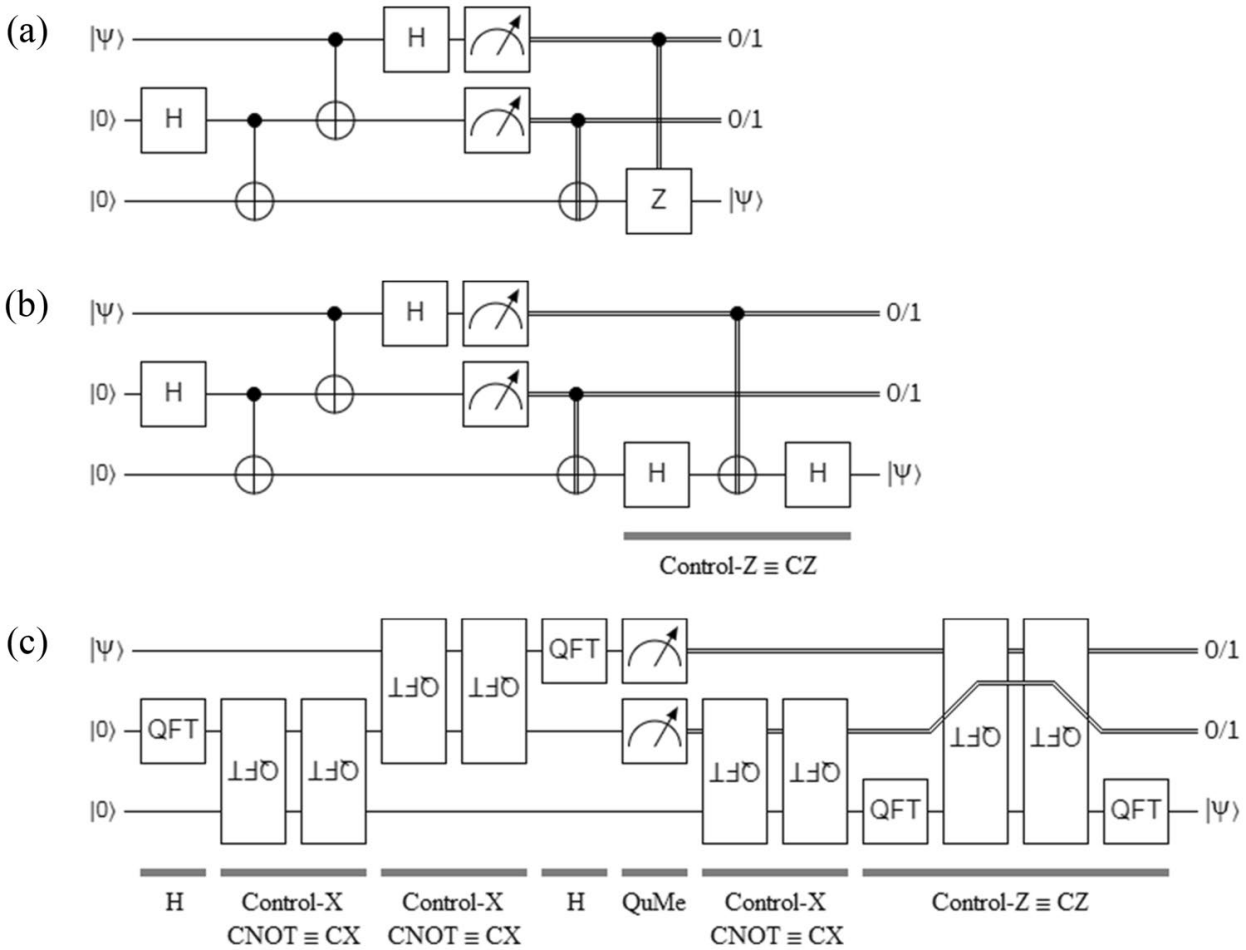
Quantum teleportation protocol: (**a**) the original implementation, (**b**) Controlled-*Z* is replaced with its equivalent in terms of one CX and two *H* gates, and (**c**) a version based exclusively on QFT blocks, where QuMe means *quantum measurement*, and the double lines represent classical information obtained after each QuMe.

This protocol^13^ is implemented in three different ways in Fig. 3, where a qubit $\left| \psi \right\rangle$ to be teleported is prepared and introduced in the upper qubit on the left of the protocol. A Bell state like that of Fig. 1 is distributed between Alice and Bob. Subsequently, a module applied in the two upper qubits and constituted by a CNOT gate, an *H* gate, and two quantum measurement blocks (QuMe) constitute what in practice is known as a Bell State Measurement (BSM) module^4,8–10^. The double lines at the output of each QuMe convey classical information from Alice to Bob in the form of two classical disambiguation or control bits. For this reason, this means of transport is known as a classic channel of disambiguation, control, or simply as an auxiliary channel.

A 2-qubits Controlled-Z gate or simply CZ gate can be constructed from QFT blocks according to the equivalence of Eq. (9d), or in terms of two *H* and one CNOT (Controlled-*X* or CX) gates from the following identity:

15 $$ CZ = \left( {I_{2 \times 2} \otimes H} \right)CX\left( {I_{2 \times 2} \otimes H} \right) $$

As can be seen in Fig. 3c, except for the QuMe blocks, everything else in this protocol is representable using QFT blocks. This extends, with identical results, to all other protocols that are based on entanglement such as quantum secret sharing^14^, quantum key distribution^15^, quantum secure direct communication^16^, and quantum repeaters^17^, and that are used in quantum Internet^18^.

### Quantum spectral analysis

**Figure 4:**
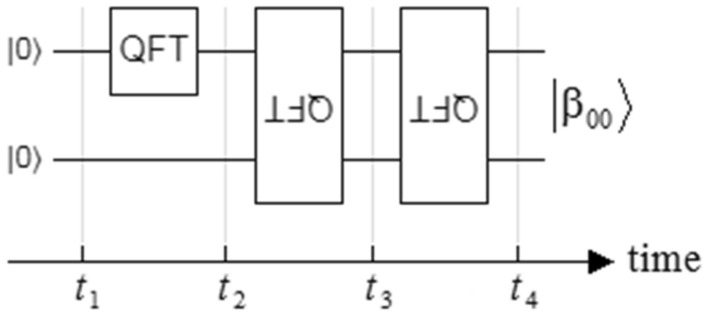
Timeline of the $\left| {\beta_{00} } \right\rangle = \left| {\Phi^{ + } } \right\rangle$ Bell state of Fig. 1b based on QFT blocks.

A time decomposition, based on Fig. 4, is developed according to the intervention of each QFT block in the creation of the $\left| {\beta_{00} } \right\rangle = \left| {\Phi^{ + } } \right\rangle$ Bell state. This analysis begins with the creation of a flipped QFT_2_^2^_×2_^2^ in terms of a QFT_2_^2^_×2_^2^ and four Hadamard (*H*) gates, where the last ones are used for flipping it as follows,

16 $$ \begin{gathered} F_{{2^{2} ,flipped}} = \left( {H \otimes H} \right)F_{{2^{2} }} \left( {H \otimes H} \right) \hfill \\ \quad \quad \quad \;{\kern 1pt} = \frac{1}{2}\left[ {\begin{array}{*{20}c} 1 & 1 & 1 & 1 \\ 1 & { - 1} & 1 & { - 1} \\ 1 & 1 & { - 1} & { - 1} \\ 1 & { - 1} & { - 1} & 1 \\ \end{array} } \right]\frac{1}{2}\left[ {\begin{array}{*{20}c} 1 & 1 & 1 & 1 \\ 1 & i & { - 1} & { - i} \\ 1 & { - 1} & 1 & { - 1} \\ 1 & { - i} & { - 1} & i \\ \end{array} } \right]\frac{1}{2}\left[ {\begin{array}{*{20}c} 1 & 1 & 1 & 1 \\ 1 & { - 1} & 1 & { - 1} \\ 1 & 1 & { - 1} & { - 1} \\ 1 & { - 1} & { - 1} & 1 \\ \end{array} } \right] = \frac{1}{2}\left[ {\begin{array}{*{20}c} 1 & 1 & 1 & 1 \\ 1 & 1 & { - 1} & { - 1} \\ 1 & { - 1} & i & { - i} \\ 1 & { - 1} & { - i} & i \\ \end{array} } \right] \hfill \\ \end{gathered} $$

Next, the complete timeline is developed according to Fig. 4, starting at *t*_1_, where $\left| {\psi \left( {t_{1} } \right)} \right\rangle$ is the wave-function in that instant,

17 $$ \left| {\psi \left( {t_{1} } \right)} \right\rangle = \left| 0 \right\rangle \otimes \left| 0 \right\rangle = \left| {00} \right\rangle = \left[ {\begin{array}{*{20}c} 1 \\ 0 \\ 0 \\ 0 \\ \end{array} } \right] = \left| {q_{u} \left( {t_{1} } \right)} \right\rangle \otimes \left| {q_{l} \left( {t_{1} } \right)} \right\rangle $$

$\left| {q_{u} \left( {t_{1} } \right)} \right\rangle = \left| 0 \right\rangle$ is the upper qubit in Fig. 4, and $\left| {q_{l} \left( {t_{1} } \right)} \right\rangle = \left| 0 \right\rangle$ is the lower qubit in that figure. The qubits obtained $\left| {q_{u} } \right\rangle$ and $\left| {q_{l} } \right\rangle$ at time *t*_1_ are *completely independents*^8^, and are used as inputs to the next step, which is made up of an *H* gate in $\left| {q_{u} } \right\rangle$ and an identity matrix in $\left| {q_{l} } \right\rangle$,

18 $$ \begin{aligned} \left| {\psi \left( {t_{2} } \right)} \right\rangle & = \left( {H \otimes I} \right)\left| {\psi \left( {t_{1} } \right)} \right\rangle = \left( {H \otimes I} \right)\left| {00} \right\rangle = \left[ {\begin{array}{*{20}c} {1/\sqrt 2 } & 0 & {1/\sqrt 2 } & 0 \\ 0 & {1/\sqrt 2 } & 0 & {1/\sqrt 2 } \\ {1/\sqrt 2 } & 0 & { - 1/\sqrt 2 } & 0 \\ 0 & {1/\sqrt 2 } & 0 & { - 1/\sqrt 2 } \\ \end{array} } \right]\left[ {\begin{array}{*{20}c} 1 \\ 0 \\ 0 \\ 0 \\ \end{array} } \right] = \left[ {\begin{array}{*{20}c} {1/\sqrt 2 } \\ 0 \\ {1/\sqrt 2 } \\ 0 \\ \end{array} } \right] \\ & = \left( {H\left| 0 \right\rangle } \right) \otimes \left| 0 \right\rangle = \left| + \right\rangle \otimes \left| 0 \right\rangle = \left| {q_{u} \left( {t_{2} } \right)} \right\rangle \otimes \left| {q_{l} \left( {t_{2} } \right)} \right\rangle , \\ \end{aligned} $$

where $$\left| + \right\rangle = \left[ {\begin{array}{*{20}c} {1/\sqrt 2 } \\ {1/\sqrt 2 } \\ \end{array} } \right]$$, $\left| {q_{u} \left( {t_{2} } \right)} \right\rangle = \left| + \right\rangle$, and $\left| {q_{d} \left( {t_{2} } \right)} \right\rangle = \left| 0 \right\rangle$. As in the previous case, Eq. (18) shows us that both qubits obtained at time *t*_2_ are also completely independent^8^. Instead, in the next step yields,

19 $$ \left| {\psi \left( {t_{3} } \right)} \right\rangle = F_{{2^{2} ,flipped}} \left| {\psi \left( {t_{2} } \right)} \right\rangle = \frac{1}{2}\left[ {\begin{array}{*{20}c} 1 & 1 & 1 & 1 \\ 1 & 1 & { - 1} & { - 1} \\ 1 & { - 1} & i & { - i} \\ 1 & { - 1} & { - i} & i \\ \end{array} } \right]\left[ {\begin{array}{*{20}c} {1/\sqrt 2 } \\ 0 \\ {1/\sqrt 2 } \\ 0 \\ \end{array} } \right] = \left[ {\begin{array}{*{20}c} {1/\sqrt 2 } \\ 0 \\ {\left( {1 + i} \right)/2\sqrt 2 } \\ {\left( {1 - i} \right)/2\sqrt 2 } \\ \end{array} } \right] \ne \left| {q_{u} \left( {t_{3} } \right)} \right\rangle \otimes \left| {q_{l} \left( {t_{3} } \right)} \right\rangle , $$

where Eq. (19) indicates that $\left| {q_{u} } \right\rangle$ and $\left| {q_{l} } \right\rangle$ at time *t*_3_ are *correlated*^8^, i.e. although this case does not result in entanglement, $\left| {\psi \left( {t_{3} } \right)} \right\rangle$ cannot be factored. In this intermediate instance, between both flipped QFT_2_^2^_×2_^2^, the impossibility of factoring can be observed, since there are no values of $\left| {q_{u} \left( {t_{3} } \right)} \right\rangle$ and $\left| {q_{l} \left( {t_{3} } \right)} \right\rangle$, such that $\left| {\psi \left( {t_{3} } \right)} \right\rangle = \left| {q_{u} \left( {t_{3} } \right)} \right\rangle \otimes \left| {q_{l} \left( {t_{3} } \right)} \right\rangle$. Undoubtedly, this constitutes advance respect to the literature on the subject in force to date, which associated the aforementioned impossibility with the intervention of the CNOT gate, as a whole, in an exclusive way, or with an inappropriate coupling between the individual contributions of *H* and CNOT gates. In consequence, this analysis makes explicit an intermediate instance to the one already known for the non-separability and indistinguishability of the states during entanglement, which is exclusively the responsibility of a particular characteristic of the Discrete Fourier Transform^1^ (DFT) and that is inherited by the QFT^3^. This characteristic refers to the fact that the DFT is a dense matrix, i.e. all its elements are different from zero, since, they are the *N* roots of the unit or twiddle factors^1^, which when they are multiplied by the input vector produce an output vector where each of its elements represents a mixture or weighted sum of the incoming vector. Finally, the intervention of the second flipped QFT_2_^2^_×2_^2^ allows obtaining the wave-function at the time *t*_4_,

20 $$ \left| {\psi \left( {t_{4} } \right)} \right\rangle = F_{{2^{2} ,flipped}} \left| {\psi \left( {t_{3} } \right)} \right\rangle = \frac{1}{2}\left[ {\begin{array}{*{20}c} 1 & 1 & 1 & 1 \\ 1 & 1 & { - 1} & { - 1} \\ 1 & { - 1} & i & { - i} \\ 1 & { - 1} & { - i} & i \\ \end{array} } \right]\left[ {\begin{array}{*{20}c} {1/\sqrt 2 } \\ 0 \\ {\left( {1 + i} \right)/2\sqrt 2 } \\ {\left( {1 - i} \right)/2\sqrt 2 } \\ \end{array} } \right] = \left[ {\begin{array}{*{20}c} {1/\sqrt 2 } \\ 0 \\ 0 \\ {1/\sqrt 2 } \\ \end{array} } \right] \ne \left| {q_{u} \left( {t_{4} } \right)} \right\rangle \otimes \left| {q_{l} \left( {t_{4} } \right)} \right\rangle , $$

where, as in the previous case, it is impossible to decompose $\left| {\psi \left( {t_{4} } \right)} \right\rangle$ into two independent states $\left| {q_{u} } \right\rangle$ and $\left| {q_{l} } \right\rangle$, that is, $\left| {\psi \left( {t_{4} } \right)} \right\rangle$ is not factorable. This gives rise to a very particular state of null spin called *entanglement*^8^.

The four density matrices associated with every wave-function of Fig. 4 are the following:

21 $$ \rho \left( {t_{1} } \right) = \left| {\psi \left( {t_{1} } \right)} \right\rangle \left\langle {\psi^{*} \left( {t_{1} } \right)} \right| = \left[ {\begin{array}{*{20}c} 1 \\ 0 \\ 0 \\ 0 \\ \end{array} } \right]\left[ {\begin{array}{*{20}c} 1 & 0 & 0 & 0 \\ \end{array} } \right] = \left[ {\begin{array}{*{20}c} 1 & 0 & 0 & 0 \\ 0 & 0 & 0 & 0 \\ 0 & 0 & 0 & 0 \\ 0 & 0 & 0 & 0 \\ \end{array} } \right], $$

where (•)^*^ is the complex conjugate of (•),

22 $$ \rho \left( {t_{2} } \right) = \left| {\psi \left( {t_{2} } \right)} \right\rangle \left\langle {\psi^{*} \left( {t_{2} } \right)} \right| = \left[ {\begin{array}{*{20}c} {1/\sqrt 2 } \\ 0 \\ {1/\sqrt 2 } \\ 0 \\ \end{array} } \right]\left[ {\begin{array}{*{20}c} {1/\sqrt 2 } & 0 & {1/\sqrt 2 } & 0 \\ \end{array} } \right] = \left[ {\begin{array}{*{20}c} {1/2} & 0 & {1/2} & 0 \\ 0 & 0 & 0 & 0 \\ {1/2} & 0 & {1/2} & 0 \\ 0 & 0 & 0 & 0 \\ \end{array} } \right], $$

23 $$ \begin{aligned} \rho \left( {t_{3} } \right) = \left| {\psi \left( {t_{3} } \right)} \right\rangle \left\langle {\psi^{*} \left( {t_{3} } \right)} \right| & = \left[ {\begin{array}{*{20}c} {1/\sqrt 2 } \\ 0 \\ {\left( {1 + i} \right)/2\sqrt 2 } \\ {\left( {1 - i} \right)/2\sqrt 2 } \\ \end{array} } \right]\left[ {\begin{array}{*{20}c} {1/\sqrt 2 } & 0 & {\left( {1 + i} \right)/2\sqrt 2 } & {\left( {1 - i} \right)/2\sqrt 2 } \\ \end{array} } \right] \\ & = \left[ {\begin{array}{*{20}c} {1/2} & 0 & {\left( {1 + i} \right)/4} & {\left( {1 - i} \right)/4} \\ 0 & 0 & 0 & 0 \\ {\left( {1 + i} \right)/4} & 0 & {i/4} & {1/4} \\ {\left( {1 - i} \right)/4} & 0 & {1/4} & { - i/4} \\ \end{array} } \right],\quad {\text{and}} \\ \end{aligned} $$

24 $$ \rho \left( {t_{4} } \right) = \left| {\psi \left( {t_{4} } \right)} \right\rangle \left\langle {\psi^{*} \left( {t_{4} } \right)} \right| = \left[ {\begin{array}{*{20}c} {1/\sqrt 2 } \\ 0 \\ 0 \\ {1/\sqrt 2 } \\ \end{array} } \right]\left[ {\begin{array}{*{20}c} {1/\sqrt 2 } & 0 & 0 & {1/\sqrt 2 } \\ \end{array} } \right] = \left[ {\begin{array}{*{20}c} {1/2} & 0 & 0 & {1/2} \\ 0 & 0 & 0 & 0 \\ 0 & 0 & 0 & 0 \\ {1/2} & 0 & 0 & {1/2} \\ \end{array} } \right]. $$

The four density matrices can be seen in Table 1, where only $\rho \left( {t_{3} } \right)$ has an imaginary part. On the other hand, comparing the 3D bars of the density matrices at *t*_2_ and *t*_4_, it can be seen that the consecutive action of both flipped QFT_2_^2^_×2_^2^ has a stretching effect as far as the locations of the bars are concerned. This shows that both flipped QFT_2_^2^_×2_^2^ are the architect of a bad copy of wave-function $\left| {\psi \left( {t_{2} } \right)} \right\rangle$ of Eq. (18), that is to say,

25 $$ U\left| {\psi \left( {t_{2} } \right)} \right\rangle = U\left( {\left| + \right\rangle \left| 0 \right\rangle } \right) \ne \left| + \right\rangle \left| + \right\rangle , $$

where *U* = flipped QFT_2_^2^_×2_^2^ × flipped QFT_2_^2^_×2_^2^, being the true result generated by both,

26 $$ U\left| {\psi \left( {t_{2} } \right)} \right\rangle = U\left( {\left| + \right\rangle \left| 0 \right\rangle } \right) = \left| {\beta_{00} } \right\rangle . $$

**Table 1:** Density matrices of the four wave-functions of Fig. 4.

Instant	Real part of the density matrices	Imaginary part of the density matrices
*t*_1_		–
*t*_2_		–
*t*_3_		
*t*_4_		–

This shows that entanglement is the result of an inadequate copy by a very inefficient copy machine embodied by both flipped QFT_2_^2^_×2_^2^. The aforementioned stretching effect added to the unification of the entanglement's own wave-function triggers its most conspicuous characteristic, that is to say, the impossibility of factoring the wave function of Eq. (20).

Moreover, given two subsystems (A, and B) that interact with each other, their density matrices treated individually are,

27 $$ \rho^{A} = \rho^{B} = \frac{1}{2}\left( {\left| 0 \right\rangle \left\langle 0 \right| + \left| 1 \right\rangle \left\langle 1 \right|} \right) = \frac{1}{2}I = \frac{1}{2}\left[ {\begin{array}{*{20}c} 1 & 0 \\ 0 & 1 \\ \end{array} } \right], $$

and their von Neumann entropies are,

28 $$ S^{A} = S^{B} = - tr\left[ {\rho^{A} log\left( {\rho^{A} } \right)} \right] = - tr\left[ {\rho^{B} log\left( {\rho^{B} } \right)} \right] = - tr\left[ {\frac{1}{2}\left[ {\begin{array}{*{20}c} 1 & 0 \\ 0 & 1 \\ \end{array} } \right]log\left( {\frac{1}{2}\left[ {\begin{array}{*{20}c} 1 & 0 \\ 0 & 1 \\ \end{array} } \right]} \right)} \right] = 1, $$

where *tr*(•) is the *trace of the square matrix* (•), and *log*(•) is *logarithm base 2 of* (•). In the same way, for a composed system, the entropy is,

29 $$ S^{A \cup B} = - tr\left[ {\rho^{A \cup B} log\left( {\rho^{A \cup B} } \right)} \right]. $$

$S^{A \cup B}$ depends on the degree of correlation (completely independent, correlated, and entangled) between both subsystems. Besides, in the classical and the quantum worlds, the correlations between the subsystems are those established by the additional information. In the case of composite quantum systems, the mutual information $S^{A \cap B}$ is introduced to quantify that additional information, allowing us to obtain the degree of correlation between both subsystems^8^,

30 $$ S^{A \cap B} = S^{A} + S^{B} - S^{A \cup B} \ge 0. $$

Therefore, the entropy of the composite system $S^{A \cap B}$ indicates that the uncertainty of a state $\rho^{A \cup B}$ is less than the two subsystems $S^{A}$ and $S^{B}$ added together.

Table 2 shows entropies in terms of the degree of correlations between both subsystems, in such a way that when $S^{A \cup B} = 2$, the entropy of the composite system $S^{A \cap B} = S^{A} + S^{B} - S^{A \cup B} = 1 + 1 - 2 = 0$, which means that both subsystems do not have mutual information, and this null degree of correlation corresponds to the case of Eq. (18) of Fig. 4 at time *t*_2_, where $\left| {\psi \left( {t_{2} } \right)} \right\rangle$ is factored into $\left| {q_{u} \left( {t_{2} } \right)} \right\rangle = \left| + \right\rangle$, and $\left| {q_{l} \left( {t_{2} } \right)} \right\rangle = \left| 0 \right\rangle$, that is, both subsystems are completely independents. Instead, when $S^{A \cup B} = 1$, the entropy of the composite system $S^{A \cap B} = S^{A} + S^{B} - S^{A \cup B} = 1 + 1 - 1 = 1$, this case corresponds to Eq. (19) at time *t*_3_ of Fig. 4, where both subsystems are correlated, i.e., $\left| {\psi \left( {t_{3} } \right)} \right\rangle$ cannot be factored in terms of $\left| {q_{u} \left( {t_{3} } \right)} \right\rangle$ and $\left| {q_{l} \left( {t_{3} } \right)} \right\rangle$. Although both subsystems (A, and B) share information and are not separable, they do not give rise to entanglement. Finally, if $S^{A \cup B} = 0$, the entropy of the composite system $S^{A \cap B} = S^{A} + S^{B} - S^{A \cup B} = 1 + 1 - 0 = 2$, that is to say, the mutual information between both subsystems is maxima. It is about the presence of entanglement corresponding to Eq. (20) at time *t*_4_ of Fig. 4, where, as in the previous case, it is impossible to decompose $\left| {\psi \left( {t_{4} } \right)} \right\rangle$ into two independent states $\left| {q_{u} } \right\rangle$ and $\left| {q_{l} } \right\rangle$, that is, $\left| {\psi \left( {t_{4} } \right)} \right\rangle$ is not factorable.

**Table 2:** Entropies in terms of the degree of correlations between both subsystems.

Degree of correlation between both subsystems	$S_{A \cup B}$	$S_{A \cap B}$	Graphic of sets
Completely independent	2	0	
Correlated	1	1	
Entangled	0	2	

## Conclusions

This study demonstrated the existing relationship between the Feynman^4^ gate, known as Controlled-*X*, CNOT, or CX, with a pair of flipped QFT_2_^2^_×2_^2^. This, added to the already known equivalence between the Hadamard gate (H), and one QFT_2_^1^_×2_^1^ matrix, gives rise to the creation of entanglement based exclusively on QFT blocks. This equivalence is extended to the creation of entanglement between more than two particles, as is the case of the states $\left| {GHZ_{3} } \right\rangle$, and $\left| {GHZ_{4} } \right\rangle$.

A representation of the famous quantum teleportation protocol^11^ based exclusively on QFT^3,4^ blocks is achieved, which highlights a clear projection of the study carried out here on the future quantum Internet^18–22^.

The decomposition of the configuration for the creation of the entanglement in QFT blocks allows, through the timeline of Fig. 4, to perform an internal tomography of the entanglement, revealing, for the first time in the literature, the three degrees of correlation between particles^8^, that is, completely independent, correlated, and entanglement, from a single configuration.

In the same process mentioned previously, it becomes evident as never before that entanglement arises from a defective copy starring both flipped QFT_2_^2^_×2_^2^.

Finally, the spectral analysis of all quantum computing and quantum communication protocols, added to the traditional temporal analysis present in the literature, will allow a better understanding of the inner nature of the entanglement, so that this new approach can help to create new and more efficient algorithms and fault tolerant protocols.
